# AL Amyloidosis: Current Treatment and Outcomes

**DOI:** 10.1155/ah/7280805

**Published:** 2025-03-03

**Authors:** Margaret Locke, Maria Nieto

**Affiliations:** Department of Cancer, Northwell Health Cancer Institute & Donald and Barbara Zucker School of Medicine at Hofstra, Lake Success, New York, USA

## Abstract

Light chain AL amyloidosis is a systemic disorder involving tissue deposition of amyloid fibrils. Often delayed in diagnosis due to nonspecific systemic symptoms, AL amyloidosis must be confirmed on tissue biopsy. Once diagnosis is made, patients can be risk stratified based on the degree of organ involvement and high-risk cytogenetic features. Currently, the only FDA-approved first-line therapy for AL amyloidosis is a combination regimen of daratumumab, cyclophosphamide, bortezomib, and dexamethasone (DaraCyborD) with a goal of achieving a very good partial response (VGPR) after 4-6 cycles of treatment. Autologous stem cell transplant can be considered in selected cases, although there is no robust evidence of superiority over chemotherapy alone. In the relapsed/refractory setting, numerous promising therapies are still under investigation including venetoclax especially for patients with translocation t (11; 14) and chimeric antigen receptor T-cell therapy (CART) targeting B-cell maturation antigen (BCMA).

**Trial Registration:** ClinicalTrials.gov identifier: NCT04270175, NCT05451771, NCT04847453, and NCT05199337

## 1. Introduction

Light chain AL amyloidosis is a disorder involving tissue deposition of amyloid fibrils made up of immunoglobulin light chains. The prognosis in AL amyloidosis is poor as the diagnosis is often delayed, and disease progression often leads to multiorgan involvement. Although studies have been limited by the rarity of AL amyloidosis, there is a burgeoning body of literature that offers novel insights that guide risk stratification and treatment planning. Here, we review the current literature regarding the diagnosis, prognosis, and growing landscape of available treatment options for AL amyloidosis.

## 2. Clinical Presentation

**Figure 1:**
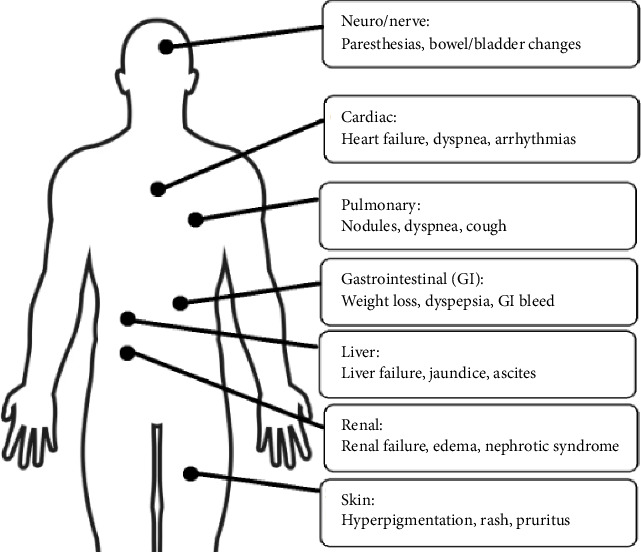
Organ manifestations of AL amyloidosis.

Part of the reason behind the often-delayed diagnosis of AL amyloidosis is the clinical presentation consisting of nonspecific systemic symptoms. Often manifesting as fatigue and unintentional weight loss, more advanced disease can present with multiorgan involvement (Figure 1). Historical studies have found that AL amyloidosis most commonly involves the heart and kidneys followed by other organs such as the liver, gastrointestinal tract, and nerves [1].

## 3. Diagnostic Approach

Evaluation of AL amyloidosis begins with a set of basic labs, including a complete CBC with differential and chemistry. Assessment for monoclonal paraprotein is performed via serum and urine protein electrophoresis. Screening a 24 h urine immunofixation electrophoresis is essential, along with serum free light chain (FLC) ratio analysis. The workup should also include quantification of proteinuria by 24-h urine collection and measurement of creatinine clearance.

The identification of FLCs in the serum or the urine must be followed by tissue confirmation of AL amyloidosis. Congo red staining of the subcutaneous fat aspirate is a reliable and noninvasive test reported to identify amyloid deposits in approximately 85% of patients. Amyloid deposits can be identified by bone marrow aspiration and biopsy followed by Congo red staining. Once amyloid is identified, amyloid typing can be confirmed by immunohistochemistry, electron microscopy, or laser microdissection with mass spectrometry–based proteomic analysis. Mass spectroscopy is considered the goal standard, and it can subtype all known subtypes of amyloidosis.

## 4. Risk Stratification

The prognosis in AL amyloidosis depends heavily on the type and extent of organ involvement as well as early diagnosis and treatment. As the heart and kidneys are the most commonly involved organs, staging schemes have been developed to quantify the degree of organ involvement.

It is well established that the degree of cardiac involvement is the biggest predictor for mortality in AL amyloidosis [1]. The revised 2012 Mayo clinic staging system uses a 4-stage system based on the cardiac biomarkers NT-proBNP and troponin as well as the difference between involved and uninvolved FLCs to identify patients at risk of early mortality. This prognostic model established disparate stages; patients with Mayo cardiac stages I, II, III, and IV achieved median overall survivals (OSs) of 94.1, 40.3, 14, and 5.8 months, respectively [2]. Other parameters such as left ventricular ejection fraction (LVEF) were also prognostic but with greater variability [1].

In addition, patients with cardiac involvement were found to have one of the longest times from symptom onset to diagnosis. This delay in diagnosis was correlated with a higher Mayo stage, reduced intensity of regimen, and greater mortality rate. Notably, a 13–18 months delay between symptom onset and diagnosis was found to have a 5-year OS of 28%, compared to 63% if diagnosis was made between 7 and 12 months of symptom onset, after accounting for lead time bias [3]. These data indicate the pressing need for timely diagnosis and treatment initiation in AL amyloidosis with cardiac involvement.

While patients with renal involvement do not share the significantly increased mortality of the patients with cardiac involvement, renal involvement carries a high degree of morbidity. The progression of AL amyloid renal disease often manifests as proteinuria and can progress to dialysis dependence. A 3-stage renal staging system was introduced by Palladini et al. based on eGFR and 24-h proteinuria. The presence of both high proteinuria (> 5 g/24 h) and eGFR < 50 mL/min portended a 60%–85% risk of dialysis dependence at 3 years [4].

Patients with renal AL amyloidosis, however, have been found to have the shortest time between symptom onset and diagnosis [1]. Epidemiological studies have found diagnosis in patients with renal involvement occurs usually within 6 months, while diagnosis in patients with other organ system involvement usually takes more than 6 months from symptom onset [5]. This difference in time to diagnosis is possibly explained by the relatively common use of urinalysis and kidney biopsy in the diagnosis of renal pathology, even if amyloidosis is not highly suspected.

Other useful prognostic markers in AL amyloidosis include bone marrow plasmacytosis, FISH cytogenetics, and karyotype. For most, poor prognosis commonly manifests as a higher rate of cardiac involvement, shorter OS, and lower hematologic response rates. Serum immunoglobulin FLCs, already a marker for assessing hematologic response, are well incorporated into cardiac staging and prognosis. In addition, > 10% bone marrow plasmacytosis carries a worse prognosis [6]. The most common cytogenetic abnormality, translocation t (11; 14) predicts inferior response to bortezomib-based regimens but instead portends a favorable prognosis in response to high dose melphalan followed by stem cell transplant (SCT) [7–9]. Data appear to be mixed about the prognostic value of gain 1q21, with a suggestion of poor prognosis in deletion 13q14, t (4; 14), t (14; 16), and deletion 17p13 [7, 8, 10, 11]. In addition, data suggest poor prognosis in trisomies with mixed data surrounding hyperdiploidy [11, 12].

## 5. Induction Therapy for Newly Diagnosed AL Amyloidosis

**Figure 2:**
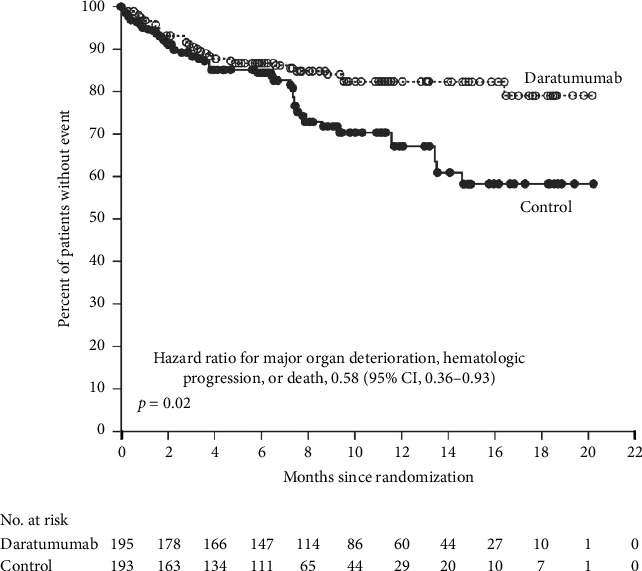
Kaplan–Meier survival curves for survival free from major organ deterioration, reproduced from the ANDROMEDA trial [13].

The only FDA–approved treatment for amyloidosis is a combination regimen of daratumumab, cyclophosphamide, bortezomib, and dexamethasone (Dara–CyborD). This follows data from the 2021 Phase III ANDROMEDA trial in patients with Stage I–IIIA AL amyloidosis. ANDROMEDA demonstrated the superiority of Dara–CyborD over CyborD alone with regard to rates of hematologic complete response (CR) (53.3% vs. 18.1%, relative risk ratio [RR]: 2.9; 95% confidence interval [CI]: 2.1–4.1; *p* < 0.001) and survival free from major organ deterioration (hazard ratio [HR]: 0.58; 95% CI: 0.36–0.93; *p*=0.02) (Figure 2), although there was no significant difference in OS [13]. This has become the current standard of care used for transplant-ineligible patients or as induction therapy prior to transplant.

The role of autologous SCT in AL amyloidosis is currently unclear, as there is currently no robust randomized trial evidence demonstrating superiority over chemotherapy alone. A Phase III randomized controlled trial evaluating melphalan followed by SCT against melphalan with dexamethasone in patients with newly diagnosed AL amyloidosis found that median OS was lower in the SCT group at 3 years follow-up (22.2 months vs 56.9 months, *p*=0.04) [14]. These data suggest potential harms and early mortality with SCT. However, a more recent head-to-head comparison of melphalan followed by SCT against melphalan and dexamethasone suggested improved 3-year OS in the SCT group (83.6% vs 58.8%), although not statistically significant [15]. With better patient selection, SCT has seen dramatic improvements in posttransplantation survival over the past decades and may be a good option for deep, sustained hematologic response in select patients [16–18]. Patients with t (11;14), for example, may have a suboptimal response to bortezomib-based induction regimens but respond well to high-dose melphalan followed by SCT [7]. Conversely, patients with t (4; 14), t (14;16), and del (17p13) have been documented to have inferior outcomes after SCT [19]. These data, in light of the potential for SCT–associated morbidity, demonstrate the growing importance of careful risk-adapted patient selection prior to SCT. With the advent of newer therapies touting more robust hematological responses such as Dara–CyborD, the role of SCT is still debated but may be selectively offered to younger patients with good performance status.

Currently, there is no universal set of criteria to determine transplant eligibility. Published selection criteria include those used by the Mayo Clinic, Boston University, and United Kingdom' National Amyloidosis Criteria with targets for performance status, age, cardiac stage, systolic blood pressure, presence of neuropathy, and markers of renal function [20–22]. In addition, although cardiac involvement had posed limitations to transplant eligibility in the past, there are data to suggest favorable outcomes in otherwise good transplant candidates with advanced cardiac involvement and/or multiorgan involvement when given dose reductions in conditioning melphalan [18, 23].

In patients with significant organ involvement, the role of solid organ transplant has been contentious. Although no randomized controlled trials have addressed this question specifically, there have been small retrospective studies that suggest a role for solid organ transplant in selected candidates. One study found that outcomes after heart transplant were similar to those of nonamyloid patients and were able to be followed by delayed SCT with good outcomes [24]. Another study found that renal transplants seemed to confer extended OS, although the best outcomes were observed in patients who had achieved hematologic CR or VGPR [25].

Hematologic response to therapy can be monitored through serum FLC levels, and organ response through organ-specific biomarkers. Routine assessments should be performed every 3 months while on active treatment. Most centers recommend achieving a minimum of very good partial response (VGPR) prior to SCT, which is defined as dFLC < 40 mg/L [26]. The inability to achieve this target after 4-6 cycles of treatment or an increase in baseline FLCs may constitute relapsed or refractory disease that requires an alternative regimen.

## 6. Relapsed/Refractory Treatments

In the relapsed/refractory setting, treatment plans should be individualized based on prior treatment exposures. In patients who had not previously received proteasome inhibitor (PI), a bortezomib-based regimen is a reasonable option [27]. Carfilzomib, another PI, is also a viable option to mitigate the risk of high-grade neuropathy associated with bortezomib [28, 29]. Ixazomib was previously studied in the Phase III TOURMALINE-AL1 trial, although it failed to meet the primary endpoint of hematological response rate [30].

Patients without prior anti-CD38 exposure warrant consideration of daratumumab. It also remains to be seen whether there is a role for other novel monoclonal antibodies targeting CD38 such as isatuximab in patients refractory to daratumumab.

Another reasonable option includes the immunomodulatory drugs (IMiDs). While IMiDs are a key component of frontline treatments for multiple myeloma, they have modest activity in AL amyloidosis. Although with limited data and no direct head-to-comparison trials between IMiDs for relapsed/refractory AL amyloidosis, pomalidomide appears to have the best response and side effect profile when compared to lenalidomide and thalidomide [31–33]. However, the promising hematological responses must be carefully considered against the high rate of toxicities, namely, cytopenias.

In patients with translocation t (11; 14), given data showing suboptimal response to bortezomib-based regimens, the BCL-2 inhibitor venetoclax may be an attractive option. In patients with multiple myeloma with translocation t (11; 14), venetoclax has demonstrated efficacy with regard to progression-free survival (PFS) in the Phase III BELLINI trial [34]. Therefore, venetoclax is also undergoing investigation for use in AL amyloidosis with promising data from small, retrospective studies [35, 36].

There have been significant innovations in chimeric antigen receptor T-cell therapy (CART), and B-cell maturation antigen (BCMA) targeting agents have also shown promising data in the relapsed/refractory setting. BCMA is a protein belonging to the tumor necrosis factor superfamily that regulates B-cell proliferation, survival, and maturation into plasma cells [37]. Anti-BCMA CART was initially studied in patients with multiple myeloma, although several trials excluded patients with AL amyloidosis due to several concerns including cytokine release syndrome (CRS), immune effector cell–associated neurotoxicity syndrome (ICANS), and that patients with amyloid-induced cardiac and renal involvement may not have enough organ reserve to safely tolerate toxicities of these therapies.

However, the use of anti-BCMA CART in AL amyloidosis has strong theoretical backing in that patients with AL amyloidosis have a high BCMA expression. Studies estimate median BCMA expression in patients with AL amyloidosis to be around 65% (range: 50–80) with no patient being reported with < 50% expression. The staining pattern was membranous in 50%, Golgi in 17%, and Golgi-membranous in 33% [38, 39]. Two case reports of AL amyloidosis treated with anti-BCMA CART have been reported in the literature. One patient with renal and cardiac involvement treated with ide-cel achieved VGPR, which he retained for over 258 days. Another patient with cardiac involvement treated with cilta-cel achieved cardiac response with a > 30% decrease in pro-BNP at 9 months post-CART [40].

The largest cohort treated with anti-BCMA CART (HB10101) reported 9 patients with AL amyloidosis, refractory to PI, IMiD, and anti-CD38 antibody, who had been treated previously with a median of 6 lines of therapy. Seven patients had cardiac involvement, including 4 with Mayo Stage 3a/3b prior to study entry. The overall hematological response was 100% with CR in 5 patients, VGPR in 2 patients, and partial response in 1 patient. Minimal residual disease (MRD) negativity on flow cytometry was achieved in 5 out of 8 patients [41]. The results demonstrated acceptable toxicity in this frail population, with a remarkable hematological efficacy and organ responses in most participating subjects [6]. The results of these studies demonstrate remarkable hematological and organ efficacy response in patients with AL amyloidosis and suggest that the anti-BCMA CART modality may become a powerful clinical tool to improve organ function and survival in these patients.

Advances over the past few decades have led to new insights into risk stratification and the development of treatments with higher efficacy and more tolerability. Although delays in diagnosis still pose a formidable challenge, there have been exciting new treatments as well as tools to risk stratify and mitigate treatment-related toxicities. With much innovation occurring in the relapsed/refractory setting, we look forward to additional improvements in care for this rare disease.

## Data Availability

The data that support the findings of this study are available from the corresponding author upon reasonable request.

## Nomenclature

AL: Amyloidosis immunoglobulin light chain amyloidosis
CART: Chimeric antigen receptor T-cell therapy
BCMA: B-cell maturation antigen
VGPR: Very good partial response
Dara–CyborD: Daratumumab, cyclophosphamide, bortezomib, and dexamethasone
FLC: Free light chain
NT-proBNP: B-type natriuretic peptide
OS: Overall survival
CR: Complete response
ASCT: Autologous stem cell transplantation
PI: Proteosome inhibitor
IMiDs: Immunomodulatory drugs
VGPR: Very good partial response
